# Incidental findings on MRI scans of patients presenting with audiovestibular symptoms

**DOI:** 10.1186/1472-6815-10-6

**Published:** 2010-06-07

**Authors:** Vasileios Papanikolaou, Mohammad H Khan, Ivan J Keogh

**Affiliations:** 1 Academic Department of Oto-Rhino-Laryngology, National University of Ireland Galway, Galway, Ireland; 2 Department of Otolaryngology, Head and Neck Surgery, Galway University Hospitals, Galway, Ireland; 3 Department of Otolaryngology, Head and Neck Surgery, Royal Victoria Eye and Ear Hospital, Dublin, Ireland

## Abstract

**Background:**

The evaluation of patients presenting with audiovestibular symptoms usually includes MRI of the internal auditory meatus, the cerebellopontine angle and the brain. A significant percentage of these scans will present unexpected, incidental findings, which could have important clinical significance.

**Objective:**

To determine the frequency and clinical significance of incidental findings on MRI scans of patients with audiovestibular symptoms.

**Materials and methods:**

A retrospective analysis of 200 serial MRI scans.

**Results:**

Gender distribution: equal. Age range: 17-82 years. One-hundred and four scans (52%) were normal and 1 scan (0.5%) demonstrated a unilateral vestibular schwannoma. Ninety-five scans (47.5%) demonstrated incidental findings. Sixty-six of these (33%) were considered of ishaemic origin and did not require further action. Five (2.5%) scans demonstrated significant findings which warranted appropriate referral; Two Gliomas (1%), 2 cases of extensive White Matter Lesions (1%), 1 lipoma (0.5%). The remaining scans demonstrated various other findings.

**Conclusion:**

Investigation of patients with audiovestibular symptoms with MRI scans revealed incidental findings in a significant percentage (47.5%). The majority of these findings were benign warranting no further action and only 2.5% required further referral. It is the responsibility of the referring Otolaryngologist to be aware of these findings, to be able to assess their significance, to inform the patient and if needed to refer for further evaluation.

## Background

Patients frequently present to the otorhinolaryngologist with audiovestibular symptoms such as; asymmetrical hearing loss, unilateral tinnitus, sudden sensorineural hearing loss and vertigo. Investigation of these patients includes thorough clinical examination, audiological evaluation and frequently Magnetic Resonance Imaging (MRI) of the Internal Auditory Meatus (IAM), cerebellopontine angle (CPA) and brain. MRI scanning is a well-established, cost-effective investigation for these patients [1-3]. Only a small percentage of these scans reveal some form of causative pathology. However, a significant percentage will present unexpected findings which may have clinical significance. MRI findings which are unrelated to the purpose of the examination are considered incidental findings [4].

A growing body of literature exists in regards to incidental findings in various groups of patients. Mirza et al reported a frequency of 41% of incidental findings while examining MRI scans for CPA tumours [5]. Katzman et al reported on incidental findings in a large group of healthy volunteers [6]. They reported a 18% occurrence of incidental findings and a 1.1% occurrence of clinically significant findings. The Cardiovascular Health Study [7,8] reported a 1.7% occurrence of clinically serious incidental findings in MRI brain scans and the Rotterdam study [9] concluded that incidental findings in the general population are quite common.

Therefore, we performed a retrospective analysis of 200 serial MRI scans, of patients with audiovestibular symptoms. The aim of our study was to define the frequency of incidental findings, their severity and clinical importance, as well as any further management needed.

## Methods

This study was performed by the Academic Department of Oto-Rhino-Laryngology, National University of Ireland, Galway and the Department of Otorhinolaryngology, Royal Victoria Eye and Ear Hospital Dublin, Ireland.

We retrospectively reviewed 200 serial MRI scans requested for patients with audiovestibular symptoms.

These patients presented to the respective outpatient departments complaining of asymmetrical hearing loss, unilateral tinnitus, sudden sensorineural hearing loss and atypical vertigo. A thorough clinical ENT examination was performed, which included a pure tone audiogram, tympanogram and clinical vestibular testing. No electophysiological and vestibular studies were performed at this stage. Additionally an MRI scan of the IAM, CPA and brain was requested, the primary aim being to exclude CPA lesions.

For this study asymmetrical hearing loss was defined as a difference of 20 dB HL or more at one frequency and 15 dB HL or more in two consecutive frequencies in bone conduction thresholds. Sudden sensorineural hearing loss was defined as a drop of 15 dB HL in three consecutive frequencies or 20 dB HL in one frequency within 72 hours or less. Patients with vertigo presenting with non-typical clinical features and disease evolution and/or with non-typical responses to vestibular testing where characterized as cases of atypical vertigo.

No ethics approval was needed for this study as the use of this imaging technique is considered as an integral part of the diagnostic approach of these patients. Patients undergoing MRI scanning had consented verbally to proceed with this examination both at the time of arranging the scan and at the time of scanning.

The standard protocol for the MRI scans performed was T2-weighted high-resolution 2-3 mm sections through the IAM and 6 mm sections through the brain. Additionally, T1-weighted sections with Gadolinium enhancement were also performed.

## Results

Two-hundred MRI serial scans were reviewed (99 females and 101 males), age range 17 to 82 years.

One hundred and four scans (52%) were reported as completely normal (Table 1).

**Table 1:** Findings from MRI scanning

Finding	Number
Normal	104 (52%)

Incidental findings-NFA	90 (45%)

Incidental findings-Clinical significant	5 (2.5%)

CPA Tumour	1 (0.5%)

**Total number**	200

(NFA = no further action)

One patient (0.5%), who presented with asymmetrical sensorineural hearing loss and unilateral tinnitus, was found to have an ipsilateral vestibular schwannoma (Table 1). The patient was referred for neurosurgical evaluation and management.

**Figure 1:**
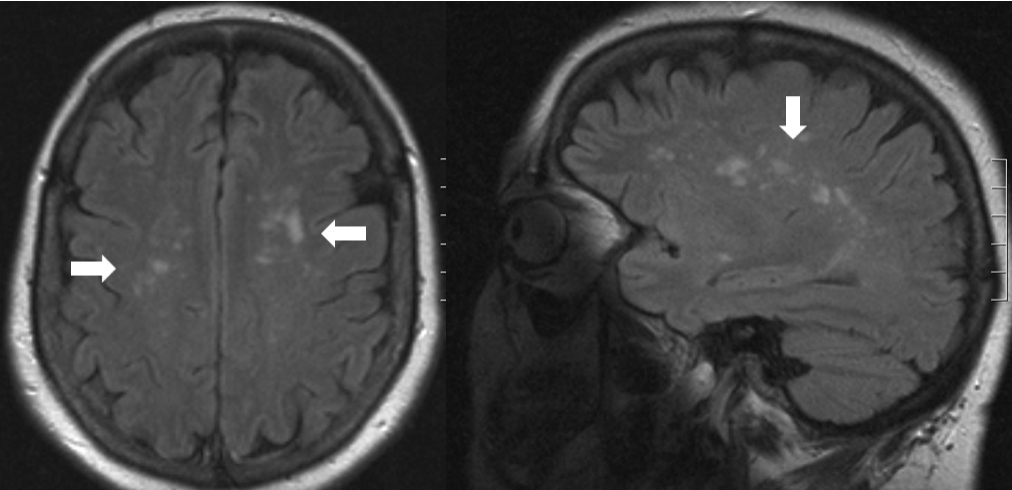
Extensive WML (horizontal and vertical cuts)

**Figure 2:**
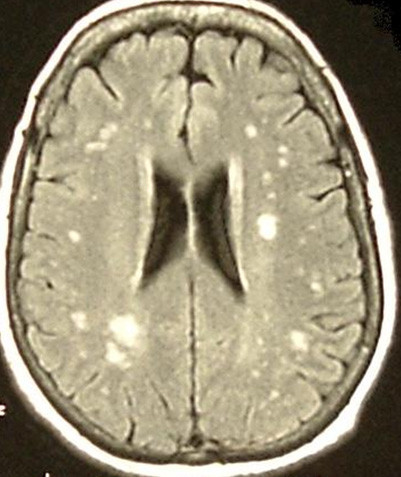
Extensive WML

Ninety-five scans (47.5%) demonstrated various incidental findings (Table 2). The most frequent incidental finding were hyperintensive white matter foci, commonly referred to as White Matter Lesions (WML). Fifty-four cases (27%) demonstrated WML alone, whereas in 14 cases (7%) the WML were noted in combination with other pathology. Of these mixed cases two patients (1%) presented extensive WML (Fig. 1, 2) and were referred to the department of neurology for further evaluation.

**Table 2:** Incidental findings from MRI scanning

Finding	Number	Percentage	Management
WML	54	27%	NFA

WML + cerebral atrophy	6	3%	NFA

Sinus findings	5	2.5%	NFA

Vascular infarcts	4	2%	NFA

WML + sinus findings	4	2%	NFA

Cerebral atrophy	3	1.5%	NFA

Middle ear/mastoid findings	3	1.5%	NFA

Glioma	2	1%	NSR

Extensive WML	2	1%	NLR

Arachnoid cyst	2	1%	NFA

WML + middle ear/mastoid findings	2	1%	NFA

Cerebral atrophy + sinus findings	2	1%	NFA

Vascular anomaly	2	1%	NFA

Lipoma	1	0.5%	NSR

Meningioma	1	0.5%	NFA

Sinus findings and Middle ear/mastoid findings	1	0.5%	NFA

Enhancement around facial nerve	1	0.5%	NFA

(NFA = no further action, NSR = Neurosurgical referral, NLR = Neurology referral)

Five patients (2.5%), had clinically serious incidental findings that needed further management.

**Figure 3:**
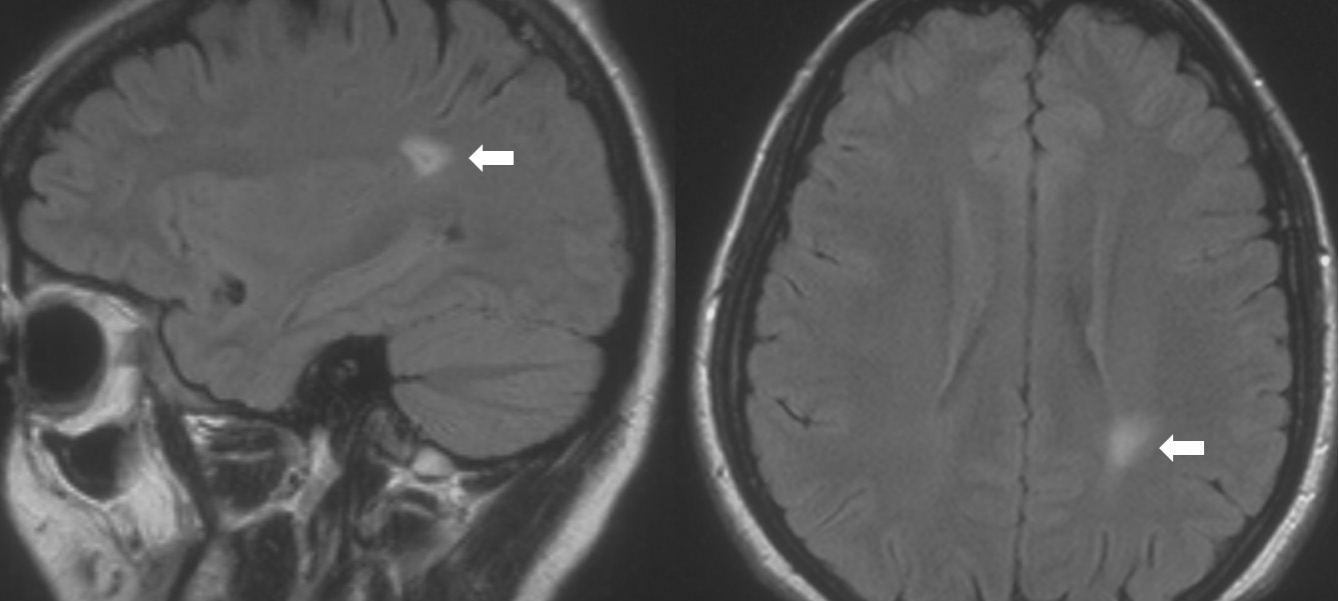
Glioma (horizontal and vertical cuts)

Two patients (1%) were found to have gliomas (one in the frontal lobe, while the other was lateral and adjacent to the posterior horn of the left lateral ventricle, (Fig. 3) and were referred to the department of neurosurgery, for further evaluation.

One patient (0.5%) was found to have a lipoma of the quadrigeminal plate cistern, 15 mm in diameter, with no noticed mass effect, and was also referred to the department of neurosurgery.

Two further patients (1%) were found to have a small arachnoid cyst (one in the temporal fossa and the other in the contralateral IAM). These were considered innocuous and no further action was warranted.

One patient (0.5%) was found to have a small meningioma arising from the vertex of the skull, contralateral to the patient's unilateral tinnitus; no further action was taken.

**Figure 4:**
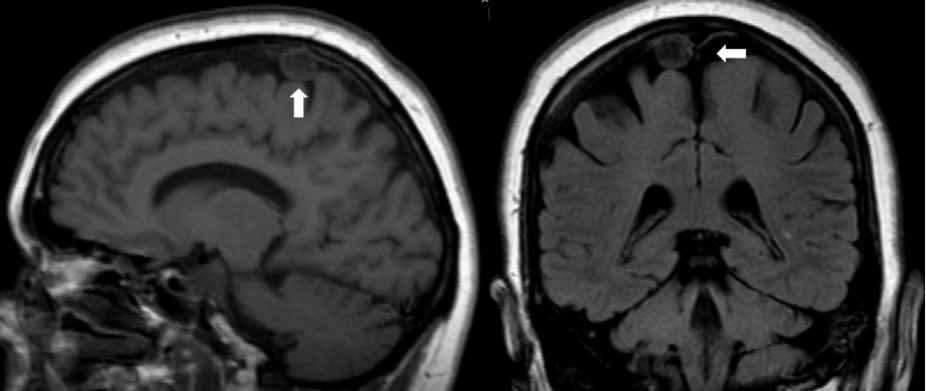
Meningioma (vertical and coronal cuts)

Three patients (1.5%) had cerebral atrophy as a sole finding, while 6 (3%) had atrophy in combination with WML (Fig. 4).

In 2 scans (1%), vascular anomalies were noted; an enlarged vascular space and a loop of the anterior inferior cerebellar artery.

Five patients (2.5%) were reported to have findings from one or multiple sinuses with findings ranging from simple mucosal thickening to pansinusitis, while 4 (2%) presented sinus findings and WML and 2 (1%) sinus findings and cerebral atrophy.

Three patients (1.5%) were reported to present high signals in the middle ear and mastoid region indicating inflammation. Another 2 (1%) presented middle ear/mastoid findings and WML and 1 (0.5%) presented both mastoid and sinus findings.

Finally 1 patient (0.5%) presented enhancement around the facial nerve, however as the patient had no complains in regards to the facial nerve or the respective middle ear no further action was warranted.

## Discussion

Our retrospective study included 200 serial scans of patients that presented with audiovestibular symptoms. Initially all patients underwent a full otolaryngologic clinical evaluation, vestibular clinical examination, pure tone audiogram and tympanogram, as suggested by the Sheppard et al investigation protocol [10]. Subsequently, they underwent an MRI scan of the IAM, CPA and brain in order to exclude any intracranial pathology.

Advanced modern MRI scanners present a high degree of accuracy in detecting vestibular schwannomas. Therefore, these sequences have been suggested as an efficient and quick method of investigation for patients with audiovestibular symptoms [11]. Additionally, a growing body of literature considers Auditory Brainstem Testing as non effective and expensive for screening [12]. Modern MRI scanners are considered to reach 100% sensitivity for acoustic tumour screening, without enhancement. However, further imaging with enhancement is needed to confirm diagnosis [12] Additionally, FLAIR (Fluid attenuation inversion recovery) sequences have been shown to be able to demonstrate inflammation of the inner ear, making MRI imaging an effective tool for the investigation of inflammatory conditions causing sudden sensorineural hearing loss [13,14]. In our study T1- weighted sections with Gadolinium enhancement and T2 weighted high-resolution 2-3 mm sections through the IAM and 6 mm sections through the brain were performed.

However, it should be noted that in some cases, MRI can not fully discover the pathological changes in the audiovestibular system, patients with negative results should be consulted in higher level hospital to get sophisticated inspection.

Approximately half of the scans (52%) performed were reported to be normal. This is a common finding in reported series of MRI scans performed in order to investigate various audiovestibular symptoms [1,5]. Studies of healthy volunteers or the general population show a higher percentage of normal scans [6,8].

In our study 96 (48%) of the scans demonstrated positive findings (Table 1). One ipsilateral vestibular schwannoma was detected in a patient with tinnitus.

The remaining 95 scans (47.5%) revealed positive findings, however these could not be directly related to the investigated symptoms, therefore they were considered as incidental findings [4]. Various studies have addressed the subject of incidental findings on MRI of the brain, as they appear to be quite common [15-23]. The Rotterdam study presented a high prevalence of incidental findings, asymptomatic brain infarcts, cerebral aneurysms and benign primary tumours being the most frequent findings [9]. Mirza et al reported in a group of patients investigated for CPA tumours a frequency of 41% of incidental findings with vascular anomalies, WML, cerebral atrophy, sinus findings and middle ear/mastoid findings being the most common [5].

In our study the most common finding was subcortical white matter hyperintensive foci, commonly referred as WML, noted either alone or in combination with other findings (68, 44%). Various studies suggest that WML are quite common, reaching a frequency of 95% in elderly patients [24]. WML appear to be age related with an increase of load and severity with age. In the Atherosclerosis Risk in Communities Study [25] their frequency increased from 88% in 55-year-olds to 92.2% in 65-year-olds. The exact nature of WML is still not defined, however currently the consensus is that they may well be ischaemic in nature. This concept has been studied in relation with various cardiovascular factors [26-29]; hypertension appearing consistently associated with their presence [30-33]. There seems to be a genetic element also to the presence of WML. Joutel at al identified mutations of the Notch 3 gene, which is located on chromosome 19, responsible for the CADASIL syndrome which presents with WML among other key features [34]. WML have been associated with various impairments of neurological function. Studies have shown WML to be associated with impairment in speed and memory function [35], motor performance, balance and gait [36]. It appears that WMLs may represent the effect of a number of pathologies and the level of their clinical significance has not been fully defined. Therefore, clinicians using MRI scans should understand the lack of specificity of this finding and assess and draw conclusions based on the patient's clinical status [37]. Due to the data collection process detailed information about the number of patients with WML and their presenting complain was not available, which is a defect of our study.

It should be stressed that a demyelinating process could also appear as hyperintensities of the white matter and therefore a differential diagnosis from an incidental WML is necessary [38]. With this in mind, extensive WML should not be dismissed as a simple incidental finding; clinical correlation and appropriate referral is warranted in such cases. Therefore the 2 patients with extensive WML that were found in our study were referred to the Department of Neurology for further evaluation.

Two of the scans revealed a large glioma, with no mass effect in either case. The lesions were large enough to warrant a referral, as there was a concern of potential haemorrhage within them.

Another patient showed a lipoma of the quadrigeminal plate cistern 12-15 mm in diameter, with no signs of mass effect on the surrounding brain structures on the MRI. However, due to the unusual presentation the patient was referred for an out-patient neurosurgical assessment.

In one case an arachnoid cyst was found in the temporal lobe and in another in the contralateral IAM. As these lesions were small, and not related to the patients' symptom (hearing loss) they were considered as benign findings [39], and therefore routine follow up was suggested.

In our study one meningioma (0.5%) was noted, in the Rotterdam study they were the most common benign tumour, with a frequency of 0.9% [9]. The majority of meningiomas appear to be asymptomatic and their growth rate is very slow [15,16]. This silent slow clinical course would explain why approximately 50% of meningiomas are discovered at autopsy [17]. In our case due to the fact that the finding was contralateral to the patient's symptoms, less than 1 cm in diameter with no clinical symptoms and taking into account the patient's age, no further action was undertaken.

Twelve patients (6%) presented findings related to their sinuses, with or without other incidental findings. Sinus findings in various MRI series performed for non-sinonasal complaints appear to be quite common. Therefore, a number of studies have been performed in order to define the clinical importance of these paranasal findings [18-20]. Most recently McNeil et al [21] in a prospective study found that there is no statistical relationship between sinonasal symptoms and MRI paranasal findings, in patients undergoing MRI scanning for non-sinonasal complaints. Currently the consensus is that these findings need to be clinically correlated when interpreting scans of the sinuses. In our series none of our patients were treated on the basis of the scan results.

Eleven patients (5.5%) presented a degree of cerebral atrophy, with or without any other findings. Scahill et al [22] observed the effect of age on global and regional brain volumes in healthy individuals. Their work showed that there is significant decrease in the cross-sectional volume of whole brain with the advance of age, a finding confirmed by our study. Kerber et al [23] demonstrated a relationship between cerebral atrophy, WML and disequilibrium, however there was no direct association with vertigo.

Two patients (1%) presented with vascular anomalies. One presented with an enlarged vascular space, which was considered an anatomical variation. The other complained of unilateral mild tinnitus, showed an ipsilateral vascular loop of the anterior inferior cerebellar artery on MRI. The loop was in close contact with the facial and vestibulocochlear nerves. Such vascular loops are not uncommon findings on various anatomical studies [40,41] and can present in close proximity to the CPA, the IAM or the vestibulocochlear nerve. Reisser et al [42] studied 1327 human temporal bones and found that 12.3% of the specimens featured an anterior inferior cerebellar artery loop, a finding that did not correlate with unexplained hearing loss, vertigo, tinnitus or Meniere's disease. Therefore, based on the available data such findings could be considered a normal anatomical variant. Conversely, a number of authors have suggested that compression of the VIII cranial nerve by vascular loops of the anterior inferior cerebellar artery may be a cause of symptoms as vertigo, tinnitus, and sensorineural hearing loss; however data in favor of this mechanism is limited [43-47]. Despite the lack of evidence a number of authors report positive results after surgical decompression of the VIII cranial nerve [48-51]. After explaining our findings, possible explanations and treatment options the patient refused any further interventions.

There were 6 patients with middle ear/mastoid region inflammation, with or without some other form of incidental finding. Four of these patients had previously undergone middle ear surgery, or were treated for chronic middle ear disease. However, as none of the patients presented active disease, no immediate action was undertaken.

Finally one patient was found to have a degree of enhancement around the facial nerve. As the patient did not present any associated symptoms, no further action was needed.

## Conclusion

The great sensitivity of the MRI as an investigative tool for the clinician allows the detection of previously undetectable pathologies and anomalies. In our retrospective evaluation of 200 MRI scans of patients with audiovestibular symptoms, incidental findings were demonstrated in 95 cases (47.5%); Ninety cases (45%) of these incidental findings were benign and warranted no further action. However, 5 cases (2.5%) demonstrated serious clinical incidental findings and required appropriate referral to other specialties. It is therefore the responsibility of the referring clinician to assess the clinical significance of the various findings, inform the patient, and refer for further evaluation, if necessary.

## Abbreviations

MRI: Magnetic Resonance Imaging; IAM: Internal Auditory Meatus; CPA: Cerebellopontine Angle; dB HL: Decibel hearing level; WML: White Matter Lesions; FLAIR: Fluid attenuation inversion recovery;

## Competing interests

The authors declare that they have no competing interests.

## Authors' contributions

VP and MK carried out the data collection and process and drafted the manuscript. IK conceived, designed and supervised the study. All authors have read and approved the final manuscript.

## Pre-publication history

The pre-publication history for this paper can be accessed here:

http://www.biomedcentral.com/1472-6815/10/6/prepub
